# 

**Published:** 1869-03

**Authors:** 


### CONTENTS.

ORIGINAL COMMUNICATIONS.

Artificial Dentures.... 97
Valedictory Address to the Graduating Class, Ohio Dental College, March 3d, 1869.... 104
The Health of the Dentist—Means of Preserving.... 108

PROCEEDINGS OF SOCIETIES.

The Twenty-fifth Annual Meeting of the Mississippi Valley Dental Association.... 121

SELECTIONS.

The Treatment of Neuralgia by Electrization.... 127
Carbolic Acid Treatment in Surgery.... 130
A Wide Circuit.... 130
The Action of Belladonna.... 131
To Ascertain the Power of a Microscope.... 133
The First Inoculation for Small-Pox.... 133
The First Quack in America.... 133
Ney's Battery.... 134
Coffee as a Deodorizer.... 134
Brown Sequard.... 134
The First Persons Vaccinated in America.... 134

EDITORIAL.

Continuous Gum Work.... 135
American Dental Association.... 138
Do we use Os-Artificial.... 140
The Physician's Dose and Symptom Book.... 140

